# Changes in Dynamics Within and Between Resting-State Subnetworks in Juvenile Myoclonic Epilepsy Occur at Multiple Frequency Bands

**DOI:** 10.3389/fneur.2018.00448

**Published:** 2018-06-14

**Authors:** Zhe Zhang, Guangyao Liu, Zhijun Yao, Weihao Zheng, Yuanwei Xie, Tao Hu, Yu Zhao, Yue Yu, Ying Zou, Jie Shi, Jing Yang, Tiancheng Wang, Jing Zhang, Bin Hu

**Affiliations:** ^1^Gansu Provincial Key Laboratory of Wearable Computing, School of Information Science and Engineering, Lanzhou University, Lanzhou, China; ^2^Department of Magnetic Resonance, Lanzhou University Second Hospital, Lanzhou, China; ^3^Department of Child Behavior Correction, Lanzhou University Second Hospital, Lanzhou, China; ^4^The Epilepsy Center of Lanzhou University Second Hospital, Lanzhou, China; ^5^CAS Center for Excellence in Brain Science and Intelligence Technology, Shanghai Institutes for Biological Sciences, Chinese Academy of Sciences, Shanghai, China

**Keywords:** juvenile myoclonic epilepsy, frequency-dependent, dynamic FC, time-varying, resting-state sub-network, CEEMDAN

## Abstract

Time-varying connectivity analyses have indicated idiopathic generalized epilepsy (IGE) could cause significant abnormalities in dynamic connective pattern within and between resting-state sub-networks (RSNs). However, previous studies mainly focused on the IGE-induced dynamic changes of functional connectivity (FC) in specific frequency band (0.01–0.08 Hz or 0.01–0.15 Hz), ignoring the changes across different frequency bands. Here, 24 patients with IGE characterized by juvenile myoclonic epilepsy (JME) and 24 matched healthy controls were studied using a data-driven frequency decomposition approach and a sliding window approach. The RSN dynamics, including intra-RSN dynamics and inter-RSN dynamics, was further calculated to investigate dynamic FC changes within and between RSNs in JME patients in each decomposed frequency band. Compared to healthy controls, JME patients not only showed frequency-dependent decrease in intra-RSN dynamics within multiple RSNs but also exhibited fluctuant alterations in inter-RSN dynamics among several RSNs over different frequency bands especially in the ventral/dorsal attention network and the subcortical network. Additionally, the disease severity had significantly negative correlations with both intra-RSN dynamics within the subcortical network and inter-RSN dynamics between the subcortical network and the default network at the lower frequency band (0.0095–0.0195 Hz). These results suggested that abnormal dynamic FC within and between RSNs in JME occurs at multiple frequency bands and the lower frequency band (0.0095–0.0195 Hz) was probably more sensitive to JME-caused dynamic FC abnormalities. The frequency subdivision and selection are potentially helpful for detecting particular changes of dynamic FC in JME.

## Introduction

Juvenile myoclonic epilepsy (JME), characterized by irregular jerks of shoulders and arms after awakening, is known as the most common idiopathic generalized epilepsy (IGE) of adolescence (1). Myoclonic, generalized tonic-clonic and absences, as three common seizure types of IGE, may be evoked either by non-specific factors, such as sleep deprivation and stress, or by specific stimuli like photic stimuli, eye-closure, praxis, and language (2). Cognitive impairments in attention, memory and language dysfunctions have also been found in JME patients (3, 4). Although substantial efforts have been made in the past decade, the fundamental pathogenesis of JME still remains largely unclear.

Recent years have witnessed a rapid growth of interest in capturing the time-varying properties of functional network connectivity (FNC) in the diseased and healthy controls (5, 6). Studies with the time-varying FNC analysis have shown that the driving mechanisms and cognitive implications of neural activity can be partly disentangled by observing functional connectivity (FC) fluctuations (7–10). In general, functional network can be divided into multiple resting-sate sub-networks (RSNs), where brain regions with functional overlapping or high correlations in regional activities are grouped together (11, 12). By conducting dynamic FC analysis, abnormalities of intrinsic functional architecture of the default network in children with absence seizure (AS) have been explored across three different seizure intervals (13), and the semiology of dyscognitive seizures in temporal lobe epilepsy has also been explained by altered dynamic FC within sub-cortical network related regions (14). Moreover, dynamic FC analyses often focus on the estimated FC states which represent the distinct and transient patterns of FC (15). State-specific FNC disruptions within and between several RSNs have also been found in patients with IGE in previous study (10). These findings implied that the dynamic FC analysis might be an efficient way to uncover specific time-varying abnormal properties of FC in IGE.

However, most previous studies mainly focused on the epilepsy-induced dynamic FC changes in routine frequency band (0.01–0.08 Hz or 0.01–0.15 Hz), this arbitrary selection of frequency band may lead to two main limitations. For one thing, this selection may neglect some important information in lower- or higher-frequency BOLD signals which are of some physiological significance (16, 17). For another, the selected band of 0.01–0.08 Hz or 0.01–0.15 Hz covers a rather broader spontaneous fluctuation range, and would mingle some potential information of physiological fluctuations in specific frequencies. Moreover, previous studies have revealed that different functional integration during neuronal process occurs in various frequency bands, which can be reflected by blood oxygen level dependent (BOLD) oscillations at different frequency bands (18, 19). Since BOLD oscillations of different frequency bands may imply different physiological signals (20), these rhythms might also have different sensitivity to specific brain regions. For example, Zuo et al. (21) investigated spontaneous oscillations across four different frequency bands, and found that the cortical structures present stronger amplitudes of oscillations at lower frequency (0.01–0.027 Hz) while the subcortical structures have stronger amplitudes of oscillations at higher frequency (0.27–0.073 Hz). Recently, frequency-dependent abnormalities of brain activity have also been found in epilepsy. A study investigating the changes of the amplitude of fluctuations in five distinct frequency ranges in IGE indicated that the prefrontal cortex showed amplitude difference in 0.01–0.027 Hz band, whereas the thalamus showed amplitude difference in 0.027–0.073 Hz band (22). Another study observed frequency-dependent alterations of local synchronization in IGE, and found the evident relationship between the aberrant high-frequency (>0.073 Hz) local synchronization and disease duration (23). Taken together, compared to the routine band, it is more rational to depict abnormalities of epilepsy at multiple frequencies and cover full frequency band.

Motivated by previous work, the present study aims to investigate whether the abnormalities of dynamic FC within and between RSNs in JME are associated with specific frequency bands. Five distinct frequency bands were derived by using a data-driven method named complete ensemble empirical mode decomposition with adaptive noise (CEEMDAN) (24). Then, sliding windows approach was applied to create dynamic FC matrices, and K-means clustering was also employed to identify different dynamic states. The variance of percentage of strong connectivity (SC) within and between RSNs across different states was further calculated and defined as intra-RSN dynamics and inter-RSN dynamics respectively. Additionally, correlation analysis was performed in the JME group to assess the relationship between the disease severity and the intra- and inter-RSN dynamics. Based on earlier studies, we hypothesized that JME patients exhibited significant frequency-dependent changes in intra-RSN dynamics and inter-RSN dynamics compared with healthy controls.

## Materials and methods

### Participants

Twenty-four patients with JME were recruited in the Epilepsy Center of Lanzhou University Second Hospital, Lanzhou, China. All the patients were diagnosed as JME based on the epilepsy classification criterion of the International League Against Epilepsy (ILAE) guidelines (25). Visual inspection with routine MRI scanning showed no structural abnormalities, and EEG showed 4–6 Hz generalized spike wave discharges in all patients. All of the patients had no history of using any type of antiepileptic drugs according to clinical enquiry. To assess seizure severity, all patients performed the national hospital seizure severity scale (NHS3) before the MRI scan. Twenty-four matched healthy controls were recruited via advertisements. Participants with the acute physical illness, substance abuse or dependence, a history of head injury resulting in loss of consciousness, and neurological or psychiatric disorders were excluded before scanning. This study was approved by the Ethics Committee of Lanzhou University Second Hospital. Written informed consent was obtained from each participant or one of his/her legal guardians. The demographic and clinical characteristics of all the participants are detailed in Table 1. There were no significant differences between the two groups in the age, handedness and gender (*P* > 0.05).

**Table 1:** Demographics and clinical characteristics of the participants.

**Characteristics**	**JME (*n* = 24) (Mean ± SD)**	**HC (*n* = 24) (Mean ± SD)**	***P-*value**
Age (years)	19.14 ± 1.35	20.04 ± 1.20	0.47^a^
Handedness (right/left)	24/0	24/0	–
Gender (males/females)	14/10	13/11	0.77^b^
NHS3 score	8.21 ± 3.425	–	–
New-onset (yes/no)	19/5	–	–
Antiepileptic drugs (yes/no)	0/24	–	–
Mean FD	0.12 ± 0.011	0.13 ± 0.013	0.37^a^

*Values represented mean ± SD*.

a *The P value were obtained by two-sample t-tests*.

b *The P value were obtained by Chi square test*.

### Data acquisition and preprocessing

All resting-state fMRI data were collected using a Siemens Verio 3.0 T scanner at Lanzhou University Second Hospital. Participants were told to keep silent and awake with eyes closed. They were also instructed not to think of anything in particular and not to move during scanning. Meanwhile, head motion and scanner noise were controlled by using foam paddings and earplugs. After the scan, each participant was asked to answer a questionnaire whether he or she had fallen asleep or opened eyes during the scanning. In this process, no participant's data had been excluded. A single-shot, gradient-recalled echo planar imaging sequence was applied to acquire the functional images (repetition time = 2,000 ms, echo time = 30 ms, slice thickness = 4 mm, slice gap = 0.38 mm, field of view = 240 × 240 mm, in-plane matrix = 64 × 64, flip angle = 90°, and 33 slices covered the whole brain). Finally, 200 functional volumes were collected for each participant.

Imaging data were preprocessed by using DPARSF (http://www.restfmri.net) based on SPM12 (http://www.fil.ion.ucl.ac.uk/spm). The main procedure of preprocessing included discard of the first 10 functional images, realignment, time-slicing and head motion correction, spatial normalization into the Montreal Neurological Institute (MNI) template, linear detrending, and regression of the nuisance covariates including six detrended head motion parameters, global mean signals, white matter signals and cerebrospinal signals. Participants with head movement over than 2 mm translation or 2° rotation were excluded. The mean frame-wise displacement (FD) of each participant (<0.3 mm) on the basis of realignment parameters was calculated and there were no significant differences between two groups.

### Overview

**Figure 1:**
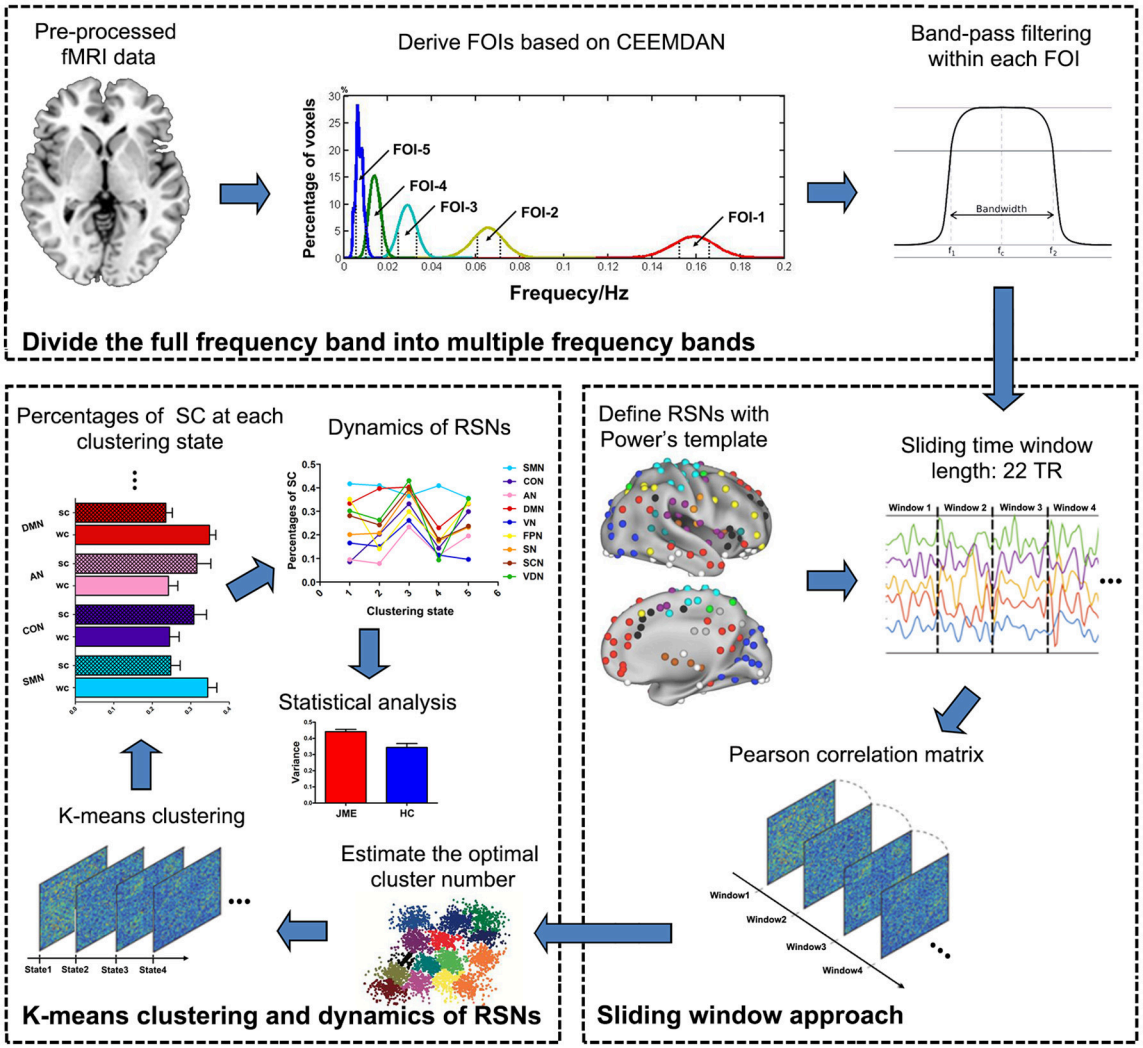
Schematic representation of the steps in frequency-dependent dynamic FC. The analysis includes three main steps as follows: (1) five FOI bands were derived with CEEMDAN method and the preprocessed resting-state fMRI data were further filtered at each FOI band; (2) the sliding windows approach was used to identify 168 time-dependent FC matrices for each participant; (3) the k-means clustering was applied to estimate reoccurring states and the dynamics of RSNs was calculated across different states.

An overview of the framework is summarized in Figure 1. Firstly, the CEEMDAN method was applied to divide the full frequency band into five distinct frequency bands and the previous preprocessed data were further filtered within each frequency band. Then, BOLD time-course from 227 brain regions located in 9 RSNs was extracted and the sliding windows approach was used to identify 168 time-dependent FC matrices for each participant. Next, the k-means clustering method on 168 window FC matrices for all participants was applied to estimate reoccurring states. Following this, the percentage of SC within and between RSNs in each state was calculated and the variance of SC across 5 states was defined as dynamics of RSNs which include intra- and inter-RSN dynamics. Finally, statistical analyses were conducted to investigate the JME-related changes in intra- and inter-RSN dynamics.

### Definition of frequencies of interest

A data-driven CEEMDAN method that can automatically decompose non-stationary signals without any rigid predefined band-pass filter was adopted to divide the BOLD signals into a finite set of distinct frequency bands (24). Briefly, a time series *x*(*t*) can be represented as $x\left(t\right)=\sum_{i\;=\;1}^{K}IMF_{i}\left(t\right)+r\left(t\right)$, where *IMF*_*i*_(*t*), *i* = 1, 2, ⋯ , *K* is a set of intrinsic mode functions, *r*(*t*) is the monotonic residue signal, and *t, i*, and *K* are the length of scanning time, the order of IMF, and the number of IMF, respectively. Note that each IMF component occupies a distinct frequency band. Specifically, the first and last IMF occupy the highest and lowest frequency band, respectively, while the remaining are in between. After decomposition, the Hilbert weighted frequency (HWF) was used to reflect the mean oscillation frequency of an IMF through instantaneous spectra information about amplitude and phase (26, 27). We calculated the HWF of each IMF in all participants and obtained the HWF distribution histograms. Then we used each HWF distribution component in 95% confidence intervals to derive a frequency of interest (FOI) for the purpose of identifing isolated frequency bands and reducing the influence of extreme values. The detailed procedure about definition of frequencies of interest was presented in our previous study (28).

### Definition of resting-state sub-networks

A brain template described by Power et al. was used in this study (29). The template contains a series of sub-networks based on the known spatial structure of resting-state fMRI network activity derived using the partitioning algorithm infomap (30). Here, we used a modified version in which 37 brain regions were excluded because of uncertainty or combination of located RSNs in Power et al. template. Thus, 227 brain regions were finally selected and categorized into 9 different RSNs, including sensory/somatomotor network (SMN, 35 regions), cingulo-opercular task control network (CON, 14 regions), auditory network (AN, 13 regions), default mode network (DMN, 58 regions), visual network (VN, 31 regions), fronto-parietal task control network (FPN, 25 regions), salience network (SN, 18 regions), subcortical network (SCN, 13 regions) and ventral/dorsal attention network (VDN, 20 regions). For each brain region, the BOLD signal intensity time-course was extracted from a sphere with a radius of 6 mm. The detailed information regarding to the parcellation of RSNs and their constitutive regions are shown in Supplementary Table S1.

### Computation for time-varying FC

For brain network analysis, brain regions can be treated as nodes of networks. Time-varying FC between all node pairs was derived using a sliding windows approach which segmented the BOLD time-course (190 TRs) into rectangular windows (22 TRs) convolved with a sigma 3-TRs of Gaussian, with the slid step of 1 TR along the length scan, resulting in 168 windows across the entire scan. Here, rectangular window length of 22 TRs was consistent with previous study, which has been proved with a good trade-off between the ability to resolve dynamics of FC and the quality of the correlation matrix estimation (15). In each sliding window, the Pearson's correlation coefficients between all node pairs were calculated to construct the covariance matrices (227 × 227) for each participant.

### Clustering analysis

After sliding windows step, k-means clustering method on 168 window FC matrices for all participants was used to estimate reoccurring FC states which represent transient patterns of FC over time (15). Specifically, k-means clustering method was first repeated 500 times to obtain several unbiased initial cluster centroids, and then these resulting cluster centroids were used as starting points to regroup all window FC matrices into one of clusters based on the similarity measurement. Here, the similarity between each FC matrix was estimated by using the L1 (Manhattan) distance function, which is an effective method for high dimensional data (31). Besides, according to previous studies (15, 32), the number of clusters input to the clustering algorithm was determined by using the elbow criterion which was calculated as the ratio of within-cluster distance to between-cluster distance. In the current study, the optimal number of clusters was determined to be five (*k* = 5). Notably, the effectiveness of the states in the JME and HC was determined according to the span of states in windows number. Meanwhile, when the state covered at least 10 windows, it was thought to be reliable state in the present study.

### Calculation of dynamics

After all FC matrices of each participant were categorized into one of five states, the percentage of SC of within and between RSNs in each state was calculated (7). For each cohort, the SC is defined as the FC with the absolute values of connectivity strength greater than the sum of average and standard deviation of this cohort. Here, we used SC to depict functional coupling between distinct brain regions because previous study has suggested that disease-related abnormalities of connectivity patterns are more prominent during SC states (32). To identify state-related connectivity strength fluctuation, we define the variances of SC that within and between RSNs across the 5 states as intra-RSN dynamics and inter-RSN dynamics respectively.

### Statistical analysis

The group differences of intra- and inter-RSN dynamics between the patients with JME and HC were evaluated using two-tailed two-sample *t*-test. The significance level was set as a threshold *P* < 0.05 with Bonferroni correction. Spearman's correlation analysis was performed between the altered intra- and inter-RSN dynamics and the NHS3 score, with age and gender regressed out. Correlations with *P* < 0.05 were considered as significant.

## Results

### Distribution of HWF in JME and HC

**Figure 2:**
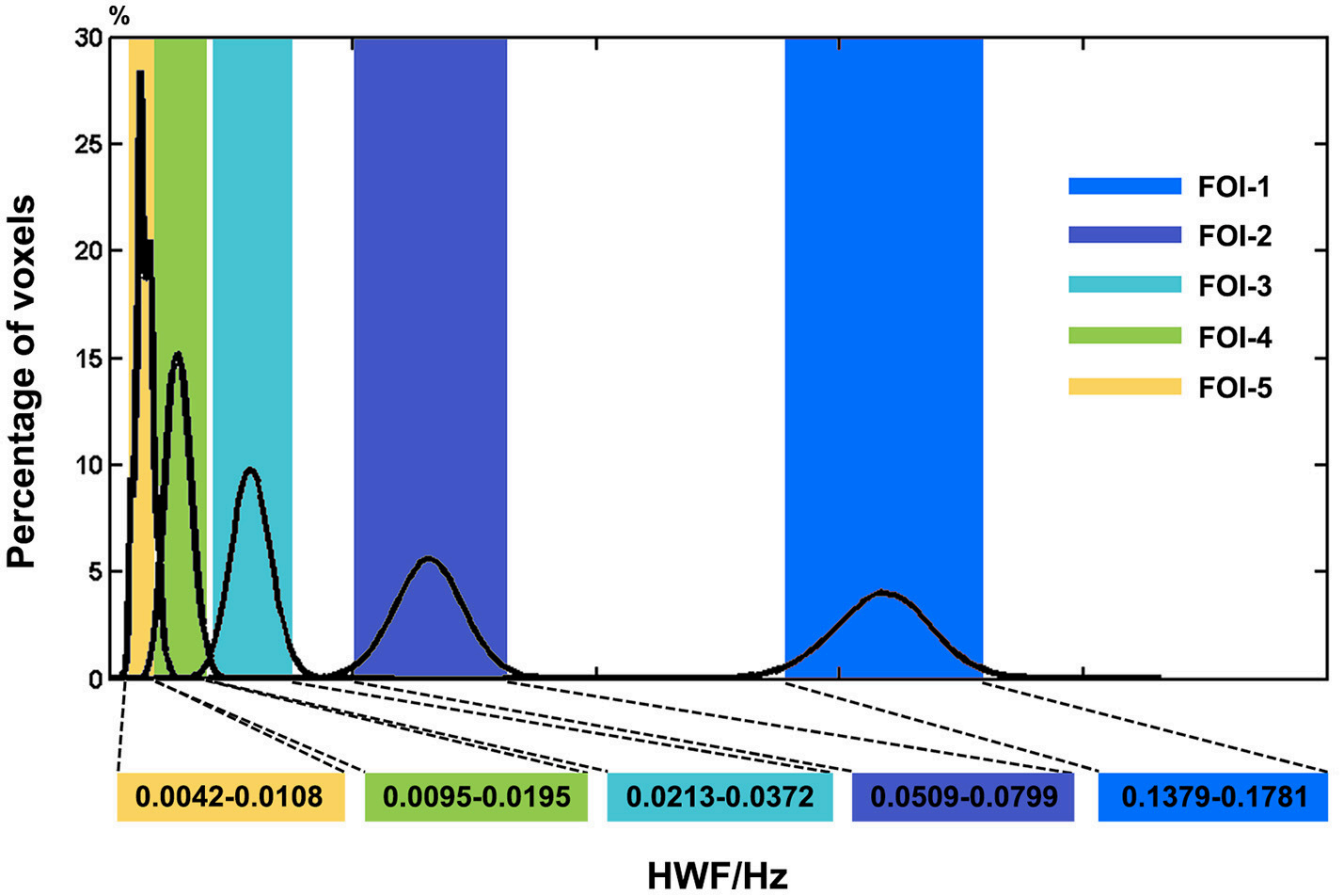
Histogram of frequency distribution of all participants. The histograms of the HWF distributions show the first five IMFs of each voxel in the whole-brain gray matter across all participants. Each of the sub-histograms in corresponding IMFs displays amount of the whole-brain gray matter voxels within all participants. Vertical axis represents the percentage of the number of voxels with HWF equal to the frequency on the horizontal axis in the whole-brain gray matter. The rectangular bar with different colors represents the frequency bands of the final selected FOIs. Colors assigned in the sequence of blue, purple, cyan, green and yellow represent FOI-1 (0.1379–0.1781 Hz), FOI-2 (0.0509–0.0799 Hz), FOI-3 (0.0213–0.0372 Hz), FOI-4 (0.0095–0.0195 Hz), and FOI-5 (0.0042–0.0108 Hz) respectively.

The histogram in Figure 2 shows the distributions of HWFs of voxels with the corresponding IMFs. Five frequency intervals (0.1379–0.1781 Hz, 0.0509–0.0799 Hz, 0.0213–0.0372 Hz, 0.0095–0.0195 Hz, and 0.0042–0.0108 Hz) were selected as FOIs to represent dynamic FC changes in JME patients. For simplicity, we numbered them from FOI-1 to FOI-5, where FOI-1 was the highest frequency interval and FOI-5 was the lowest. The percentage indicates the ratio of voxels that possesses the HWFs with the corresponding IMFs. We found that CEEMDAN could adaptively decompose the BOLD signals into several intrinsic oscillatory modes within distinct frequency bands.

### Frequency-dependent intra-RSN dynamics alterations in JME

**Figure 3:**
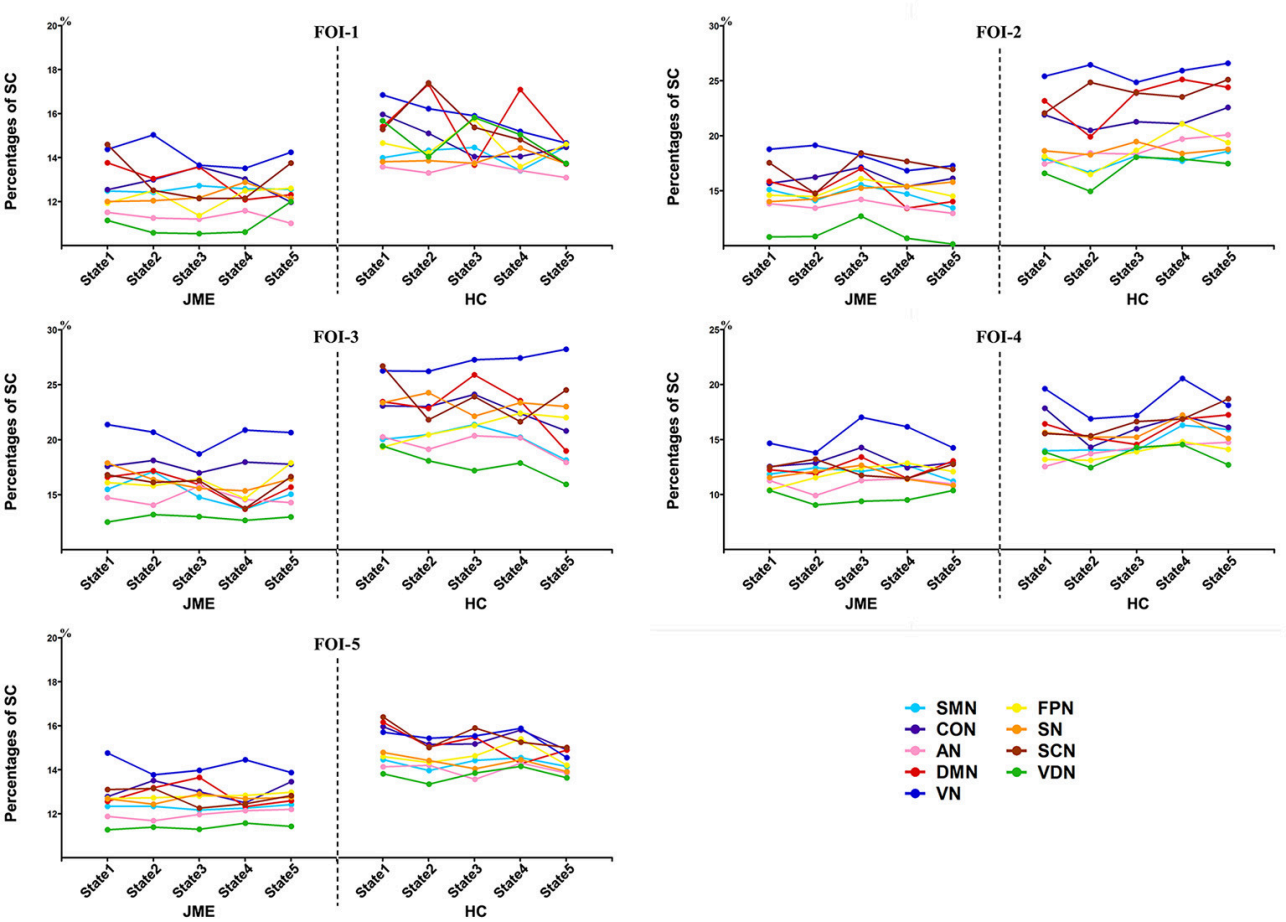
The percentages of SC within RSNs in each state across different frequency bands.

**Figure 4:**
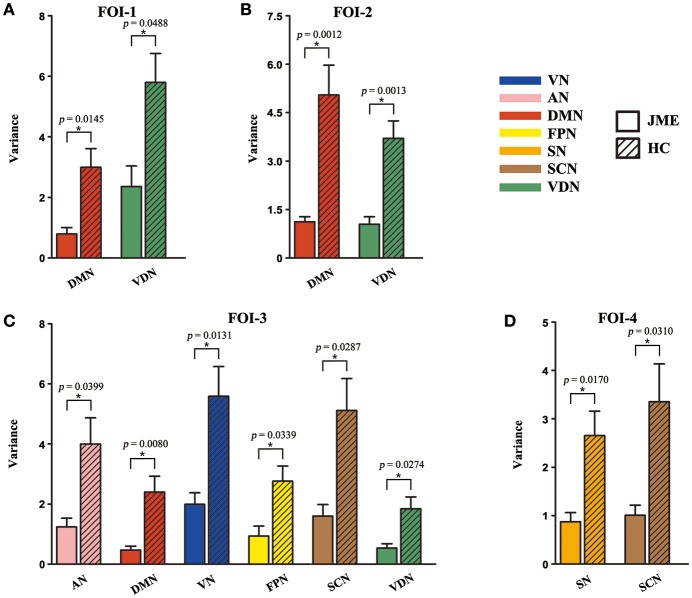
Significant between-group differences in intra-RSN dynamics across FOI-1 **(A)**, FOI-2 **(B)**, FOI-3 **(C)** and FOI-4 **(D)**. Intra-RSN dynamics with significant group differences in each FOI are depicted for JME and HC with error bars. Asterisks indicate a significant group difference with two sample *t-*test, significant level was set at *P* < 0.05 for Bonferroni corrected.

The percentages of SC within RSNs of all five states at each FOI were presented in Figure 3. We found that the percentages of SC within RSNs across all state were significantly lower in the JME group compared to healthy control. Besides, the intra-RSN dynamics across all five states was computed and a significant between-group difference was observed in FOI-1, FOI-2, FOI-3, and FOI-4, but not in FOI-5. There was indeed a frequency dependency in alterations of intra-RSN dynamics for several RSNs (Figure 4, *P* < 0.05, two tailed, Bonferroni corrected). In addition, the intra-RSN dynamics of all RSNs with between-group differences were significantly decreased in the JME group when compared with HC. To be specific, both the DMN and VDN showed decreased intra-RSN dynamics within FOI-1, FOI-2, and FOI-3; the AN, VN and FPN exhibited decreased intra-RSN dynamics only within FOI-3; the SCN displayed decreased intra-RSN dynamics within FOI-3 and FOI-4; the SN showed decreased intra-RSN dynamics only within FOI-4.

### Frequency-dependent inter-RSN dynamics alterations in JME

**Figure 5:**
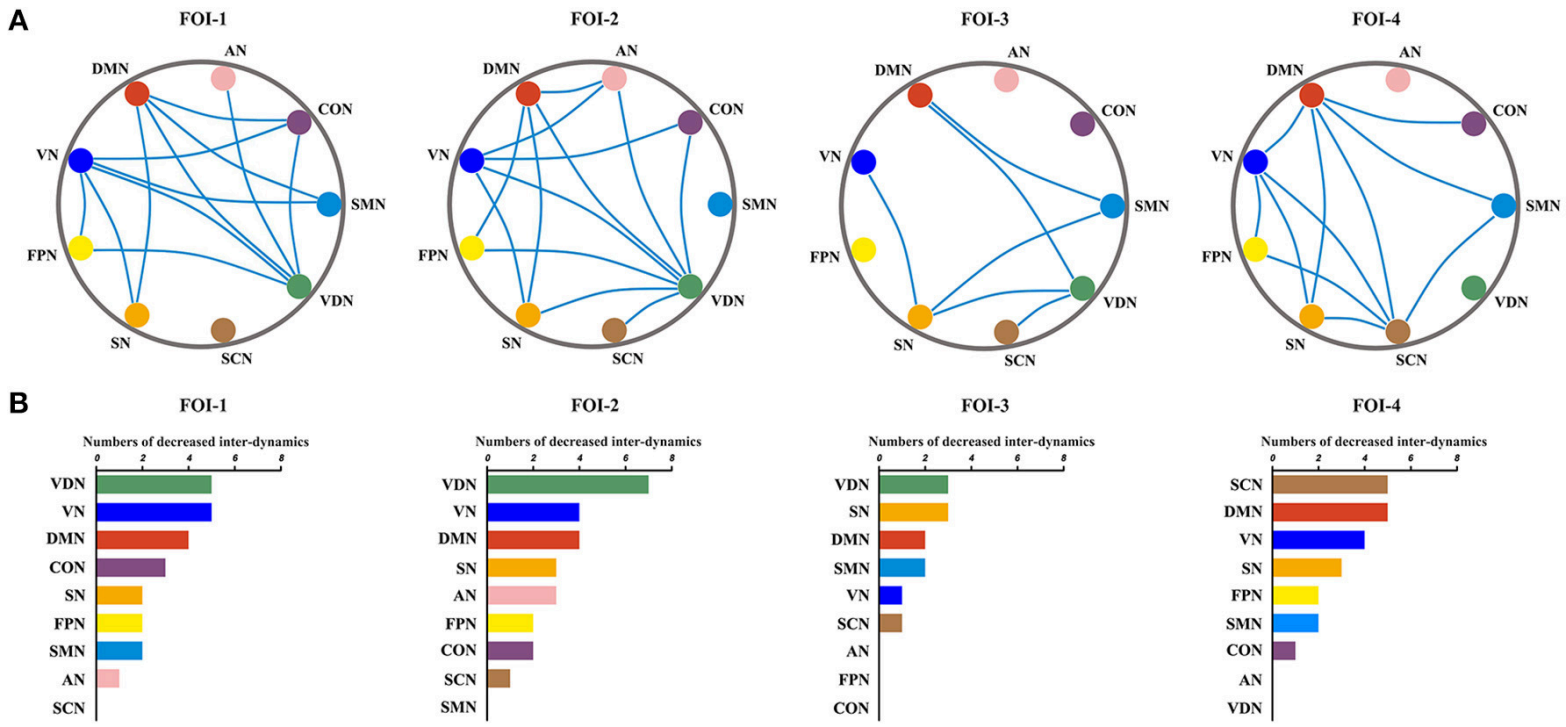
Fluctuations of inter-RSN dynamics over frequency bands. Significant between-group differences of inter-RSN dynamics **(A)** and the corresponding difference numbers of individual RSNs to the rest of RSNs **(B)** in each FOI. The blue lines represent significantly decreased inter-RSN dynamics between two pairs of RSNs in JME. Two sample *t-*test, significant level was set at *P* < 0.05, with Bonferroni corrected.

The significant between-group differences in inter-RSN dynamics were shown in Figure 5. Compared to healthy control group, the JME showed significant decrease in inter-RSN dynamics across frequency bands (*P* < 0.05, Bonferroni corrected, the detailed *P* values are shown in Supplementary Table S2). We also found that the inter-RSN dynamics of RSNs with significant group differences were fluctuant over FOI (Figure 5A, *P* < 0.05, two tailed, Bonferroni corrected). It is noteworthy that the VDN showed the most of inter-RSN dynamics differences compared with other RNSs within FOI-1, FOI-2, and FOI-3 but not within FOI-4, whereas the SCN possessed the most of inter-RSN dynamics differences within FOI-4 but not within others. The corresponding statistical results within each FOI are given in Figure 5B.

### Relationship between frequency-dependent dynamics and NHS3 score

**Figure 6:**
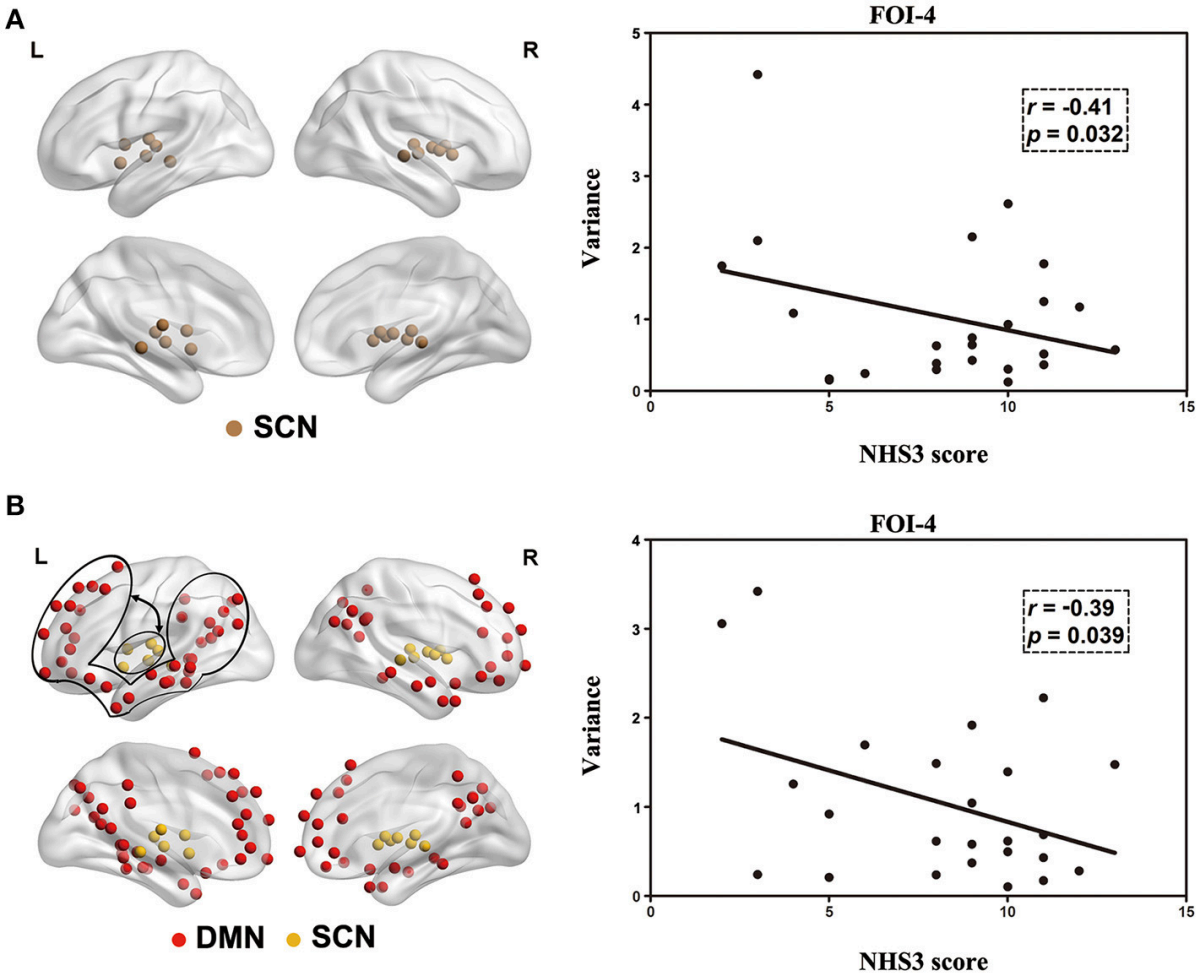
Correlation between the NHS3 score and intra-RSN dynamics **(A)** and inter-RSN dynamics **(B)** in patients with JME. Double arrows represents inter-RSN dynamics between two circled RSNs.

As shown in Figure 6A, NHS3 score was negatively correlated with intra-RSN dynamics in the SCN within FOI-4. We also computed the correlation between NHS3 score and inter-RSN dynamics of RSNs with significant inter-group differences, and found a negative correlation with inter-RSN dynamics between the SCN and the DMN within FOI-4 (Figure 6B).

## Discussion

The present study investigates the changes in dynamic FC within and between RSNs in JME at different frequency bands. Our key finding is that JME patients showed frequency-dependent decrease in intra-RSN dynamics and obvious reduction with fluctuation in inter-RSN dynamics over different frequency bands. Additionally, our result about significant correlations between the NHS3 and decreased dynamics in lower frequency band suggest JME may have specific sensitivity to this frequency band, which provides new insights into our understanding of the neurophysiological mechanisms underlying JME.

Altered temporal dynamics of RSN across discrete states has been found in other diseases, such as schizophrenia (33), autism (7), and Parkinson's disease (8, 34). However, most of these studies focused on the disrupted intra-network FC within several RSNs, few of them investigated altered inter-network FC between RSNs. In the present study, we evaluated the time-varying FNC both within and between RSNs. K-means clustering method was applied to cluster FC matrices derived from each window into five different dynamic states and the percentages of SC within and between RSNs were further calculated in each state. Our study revealed that the percentages of strong functional connectivity in all RSNs decreased in each state in JME patients, suggesting the deficient connectivity strength in JME.

Abnormalities within RSNs have been consistently reported in epilepsy patients in previous studies (35–42). In the current study, we noted significant reduction in intra-RSN dynamics including the DMN, VDN, AN, VN, FPN, SCN, and SN of JME patients. The identified intra-RSN dynamics abnormality within the DMN, known to play a critical role in self-awareness and consciousness maintenance (43, 44), may indicate impaired consciousness processing in JME (45). The VDN, FPN, SCN, and SN are involved in several cognitive processes including attention, memory, as well as consciousness, and their disturbance may be associated with the interruption of attention information processing and the memory dysfunction in JME (39, 46, 47). Moreover, the sensory perception processing systems (e.g., VN and AN) that are responsible for the interaction with external environment are also known to be deficient in JME patients (36). Taken together, our results are consistent with previous studies, suggesting that the cognitive deficits observed in JME may be related to the observed decreased intra-RSN dynamics. More importantly, the between-group differences in intra-RSN dynamics varied across different frequency bands, suggesting the frequency-dependent alteration that the altered FC within RSNs might be tailored to specific frequency bands (48, 49). In the present study, we extended current research to temporal dynamic domain and found that the abnormal dynamic FC within RSNs of JME might also be dominated by specific frequency bands. Moreover, the reduction of intra-RSN dynamics within the SCN in FOI-3 and FOI-4, and within the SN only in the FOI-4 also suggests that abnormal intra-RSN dynamics of JME may have different sensitivity toward different frequency bands.

The between-group differences of inter-RSN dynamics were fluctuant across different frequency bands, especially in the VDN and SCN. The VDN is involved in human attention processing and can be divided into two sub-systems, including the ventral attention network and the dorsal attention network (50). The ventral attention network composed of the right temporal-parietal junction and the right ventral frontal cortex always shows increased functional activity when involved in reorienting attention response to salient sensory stimuli, whereas the dorsal attention network consisting of the bilateral intraparietal sulcus and the bilateral frontal eye field shows increased functional activity when involving top-down orienting attention response to salient sensory stimuli, especially in some higher-order cognitive tasks, such as working memory and executive tasks (51). Functional impairments of these two sub-systems in epilepsy have been reported in earlier studies (41, 52, 53). The alterations in VDN-related inter-RSN dynamics may be associated with the attention dysfunction in patients with JME. The SCN, including thalamus, amygdala, hippocampus, caudate, and putamen, is highlighted in cognitive processes such as memory and information monitoring (54), and is reported with both structural and functional abnormalities in JME (4, 55–57). Moreover, abnormal FC between the SCN and the DMN in the severe epilepsy of Lennox-Gastaut syndrome has been found in a recent study (58). Another study based on static inter-network FC analysis showed that FC between the regions within SCN and several cortical RSNs was disrupted in focal epilepsies (59), and the typical absence seizures influenced FC between the SCN and cortical regions (e.g., frontal cortex and primary visual regions) that are key parts to attention and primary information processing circuit (60). These findings indicated that the execution and control function in large-scale cortical networks might be carefully modulated by the SCN and this modulation was disturbed in epilepsy (59). Therefore, the decreased inter-RSN dynamics observed between the SCN and other RSNs may reflect the disturbed modulation function, and result in cognitive deficits, such as memory and consciousness in JME patients. Since a previous study suggested that connectivity strength over different states was consistently fluctuant in brain (61), our identified inter-RSN variance of SC also may provide better understanding of transient patterns of state in dynamic FC.

NHS3 as a measure of evaluating seizure severity is valid and easily applicable. It can be used to assess seizure severity in a manner compatible with the subjective impression of people with epilepsy (62). To assess whether the frequency-dependent alteration can be as a biomarker for JME, we tested correlations between RSN dynamics and NHS3 scores. The decreased intra-RSN dynamics within the SCN in FOI-4 was significantly negatively correlated with NHS3 scores, and the decreased inter-RSN dynamics between the SCN and the DMN in FOI-4 also showed negative correlation with NHS3 scores. These significant correlations between the NHS3 and the RSN dynamics in specific frequency band indicated that the dynamics of FC in specific frequency band might be implicated in the progression of seizures in JME, and could be a potential marker for distinguishing JME patients from healthy controls. Additionally, the negative correlations that can be observed only in FOI-4 may represent the frequency-specific symptom of JME, which are in good accordance with previous study that showed Slow-5 (0.01–0.027 Hz), the overlapping of FOI-4, may play an important role in disease diagnosis and progression monitoring in JME patients (22). Our results also implicate that the FOI-4 (0.0095-0.0195 Hz) might serve as a particular frequency band in identifying abnormalities in JME patients.

There are several limitations that need to be addressed in future work. First, a relatively lower repetition times (2 TR) was used to acquire resting-state data, and this temporal resolution was limited to detect dynamics of fluctuation in higher frequency (>0.25 Hz). Thus, it would be beneficial to use a higher sampling frequency in the future investigation. Second, previous studies indicated that the head motion could significantly affect the FNC (63). Although the estimated head motion parameters including six motion parameters and mean FD were regressed out from the observed dynamic measures by implementing linear regression, it could not entirely remove the potential effects of non-linear factors on the results. The impact of head motion should be estimated in the future. Third, the EEG signal was not simultaneously recorded during the fMRI data acquisition, which may cause a missing of detecting potential effect on FC in the ictal state (64). In future studies, it would be important to evaluate the effect on dynamic FC of the interictal epileptic discharges by using simultaneous EEG-fMRI. Finally, the sample sizes utilized in the current study is relatively small, the replication study on larger sample sizes needs to be performed in the future to verify our findings.

In summary, the present study revealed that the abnormalities in dynamic FC within and between RSNs in JME were frequency dependent. Furthermore, both decreased intra-RSN dynamics and inter-RSN dynamics related to the disease severity also imply that the subcortical network might be a potential marker for disease progression, and the lower frequency band (0.0095–0.0195 Hz) might play a key role in detecting specific symptom in JME. These findings provide a new perspective on JME and may help improve our understanding of the BOLD oscillations in JME.

## Author contributions

ZZ designed the study and performed statistical analyses, drafted the manuscript, and approved the final manuscript as submitted. GL performed the MRI data acquisition and the neuropsychological assessment, statistical analyses, drafted the manuscript, and approved the final manuscript as submitted. ZY, WZ, YX, TH, YZh, YY, YZo, JS, and JY coordinated and carried out the data collection, revised the manuscript, and approved the final manuscript as submitted. TW collected the subject and approved the final manuscript as submitted. BH and JZ conceptualized the study, critically reviewed the manuscript, and approved the final manuscript as submitted.

### Conflict of interest statement

The authors declare that the research was conducted in the absence of any commercial or financial relationships that could be construed as a potential conflict of interest.

## Abbreviations

AN: auditory network
AS: absence seizure
CEEMDAN: complete ensemble empirical mode decomposition with adaptive noise
CON: cingulo-opercular task control network
DMN: default mode network
FC: functional connectivity
FNC: functional network connectivity
FOI: frequency of interest
FPN: fronto-parietal task control network
HWF: Hilbert weighted frequency
IGE: idiopathic generalized epilepsy
IMF: intrinsic mode functions
JME: juvenile myoclonic epilepsy
NHS3: national hospital seizure severity scale
RSN: resting-state sub-network
SC: strong connectivity
SCN: subcortical network
SMN: sensory/somatomotor network
SN: salience network
VDN: ventral/dorsal attention network
VN: visual network.
