# Supplementary material for: Changes in Dynamics Within and Between Resting-State Subnetworks in Juvenile Myoclonic Epilepsy Occur at Multiple Frequency Bands
Source: Front Neurol. 2018 Jun 14;9:448. doi: 10.3389/fneur.2018.00448 (PMC6010515; doi:10.3389/fneur.2018.00448)
Supplement: Supplementary file 1 [file Table_1.DOCX]

Supplementary Material

Frequency-dependent dynamics alterations of resting-state

sub-networks in juvenile myoclonic epilepsy

Zhe Zhang, Guangyao Liu, Zhijun Yao, Weihao Zheng, Yuanwei Xie, Tao Hu, Yu Zhao, Yue Yu, Ying Zou, Jie Shi, Jing Yang, Tiancheng Wang, Jing Zhang* and Bin Hu*

*** Correspondence:** Bin Hu, [bh@lzu.edu.cn](mailto:bh@lzu.edu.cn); Jing Zhang, [lztong2001@163.com](mailto:lztong2001@163.com)

# Supplementary Tables

**Supplementary Table S1. The parcellation of RSNs and their constitutive regions.**

| **ROI** | **Power et. al. ROI** | **MNI space** | | | **Radius** | **Master Assignment** | **Color** | | **Resting-state Subnetwork (RSN)** |
| --- | --- | --- | --- | --- | --- | --- | --- | --- | --- |
|  |  | **X** | **Y** | **Z** |  |  |  | |  |
| 1 | 13 | -7 | -52 | 61 | 6 | 1 |  | Cyan | Sensory/somatomotor Network |
| 2 | 14 | -14 | -18 | 40 | 6 | 1 |  | Cyan | Sensory/somatomotor Network |
| 3 | 15 | 0 | -15 | 47 | 6 | 1 |  | Cyan | Sensory/somatomotor Network |
| 4 | 16 | 10 | -2 | 45 | 6 | 1 |  | Cyan | Sensory/somatomotor Network |
| 5 | 17 | -7 | -21 | 65 | 6 | 1 |  | Cyan | Sensory/somatomotor Network |
| 6 | 18 | -7 | -33 | 72 | 6 | 1 |  | Cyan | Sensory/somatomotor Network |
| 7 | 19 | 13 | -33 | 75 | 6 | 1 |  | Cyan | Sensory/somatomotor Network |
| 8 | 20 | -54 | -23 | 43 | 6 | 1 |  | Cyan | Sensory/somatomotor Network |
| 9 | 21 | 29 | -17 | 71 | 6 | 1 |  | Cyan | Sensory/somatomotor Network |
| 10 | 22 | 10 | -46 | 73 | 6 | 1 |  | Cyan | Sensory/somatomotor Network |
| 11 | 23 | -23 | -30 | 72 | 6 | 1 |  | Cyan | Sensory/somatomotor Network |
| 12 | 24 | -40 | -19 | 54 | 6 | 1 |  | Cyan | Sensory/somatomotor Network |
| 13 | 25 | 29 | -39 | 59 | 6 | 1 |  | Cyan | Sensory/somatomotor Network |
| 14 | 26 | 50 | -20 | 42 | 6 | 1 |  | Cyan | Sensory/somatomotor Network |
| 15 | 27 | -38 | -27 | 69 | 6 | 1 |  | Cyan | Sensory/somatomotor Network |
| 16 | 28 | 20 | -29 | 60 | 6 | 1 |  | Cyan | Sensory/somatomotor Network |
| 17 | 29 | 44 | -8 | 57 | 6 | 1 |  | Cyan | Sensory/somatomotor Network |
| 18 | 30 | -29 | -43 | 61 | 6 | 1 |  | Cyan | Sensory/somatomotor Network |
| 19 | 31 | 10 | -17 | 74 | 6 | 1 |  | Cyan | Sensory/somatomotor Network |
| 20 | 32 | 22 | -42 | 69 | 6 | 1 |  | Cyan | Sensory/somatomotor Network |
| 21 | 33 | -45 | -32 | 47 | 6 | 1 |  | Cyan | Sensory/somatomotor Network |
| 22 | 34 | -21 | -31 | 61 | 6 | 1 |  | Cyan | Sensory/somatomotor Network |
| 23 | 35 | -13 | -17 | 75 | 6 | 1 |  | Cyan | Sensory/somatomotor Network |
| 24 | 36 | 42 | -20 | 55 | 6 | 1 |  | Cyan | Sensory/somatomotor Network |
| 25 | 37 | -38 | -15 | 69 | 6 | 1 |  | Cyan | Sensory/somatomotor Network |
| 26 | 38 | -16 | -46 | 73 | 6 | 1 |  | Cyan | Sensory/somatomotor Network |
| 27 | 39 | 2 | -28 | 60 | 6 | 1 |  | Cyan | Sensory/somatomotor Network |
| 28 | 40 | 3 | -17 | 58 | 6 | 1 |  | Cyan | Sensory/somatomotor Network |
| 29 | 41 | 38 | -17 | 45 | 6 | 1 |  | Cyan | Sensory/somatomotor Network |
| 30 | 255 | 47 | -30 | 49 | 6 | 1 |  | Cyan | Sensory/somatomotor Network |
| 31 | 42 | -49 | -11 | 35 | 6 | 1 |  | Cyan | Sensory/somatomotor Network |
| 32 | 43 | 36 | -9 | 14 | 6 | 1 |  | Cyan | Sensory/somatomotor Network |
| 33 | 44 | 51 | -6 | 32 | 6 | 1 |  | Cyan | Sensory/somatomotor Network |
| 34 | 45 | -53 | -10 | 24 | 6 | 1 |  | Cyan | Sensory/somatomotor Network |
| 35 | 46 | 66 | -8 | 25 | 6 | 1 |  | Cyan | Sensory/somatomotor Network |
| 36 | 47 | -3 | 2 | 53 | 6 | 2 |  | Purple | Cingulo-opercular Task Control Network |
| 37 | 48 | 54 | -28 | 34 | 6 | 2 |  | Purple | Cingulo-opercular Task Control Network |
| 38 | 49 | 19 | -8 | 64 | 6 | 2 |  | Purple | Cingulo-opercular Task Control Network |
| 39 | 50 | -16 | -5 | 71 | 6 | 2 |  | Purple | Cingulo-opercular Task Control Network |
| 40 | 51 | -10 | -2 | 42 | 6 | 2 |  | Purple | Cingulo-opercular Task Control Network |
| 41 | 52 | 37 | 1 | -4 | 6 | 2 |  | Purple | Cingulo-opercular Task Control Network |
| 42 | 53 | 13 | -1 | 70 | 6 | 2 |  | Purple | Cingulo-opercular Task Control Network |
| 43 | 54 | 7 | 8 | 51 | 6 | 2 |  | Purple | Cingulo-opercular Task Control Network |
| 44 | 55 | -45 | 0 | 9 | 6 | 2 |  | Purple | Cingulo-opercular Task Control Network |
| 45 | 56 | 49 | 8 | -1 | 6 | 2 |  | Purple | Cingulo-opercular Task Control Network |
| 46 | 57 | -34 | 3 | 4 | 6 | 2 |  | Purple | Cingulo-opercular Task Control Network |
| 47 | 58 | -51 | 8 | -2 | 6 | 2 |  | Purple | Cingulo-opercular Task Control Network |
| 48 | 59 | -5 | 18 | 34 | 6 | 2 |  | Purple | Cingulo-opercular Task Control Network |
| 49 | 60 | 36 | 10 | 1 | 6 | 2 |  | Purple | Cingulo-opercular Task Control Network |
| 50 | 61 | 32 | -26 | 13 | 6 | 3 |  | Pink | Auditory Network |
| 51 | 62 | 65 | -33 | 20 | 6 | 3 |  | Pink | Auditory Network |
| 52 | 63 | 58 | -16 | 7 | 6 | 3 |  | Pink | Auditory Network |
| 53 | 64 | -38 | -33 | 17 | 6 | 3 |  | Pink | Auditory Network |
| 54 | 65 | -60 | -25 | 14 | 6 | 3 |  | Pink | Auditory Network |
| 55 | 66 | -49 | -26 | 5 | 6 | 3 |  | Pink | Auditory Network |
| 56 | 67 | 43 | -23 | 20 | 6 | 3 |  | Pink | Auditory Network |
| 57 | 68 | -50 | -34 | 26 | 6 | 3 |  | Pink | Auditory Network |
| 58 | 69 | -53 | -22 | 23 | 6 | 3 |  | Pink | Auditory Network |
| 59 | 70 | -55 | -9 | 12 | 6 | 3 |  | Pink | Auditory Network |
| 60 | 71 | 56 | -5 | 13 | 6 | 3 |  | Pink | Auditory Network |
| 61 | 72 | 59 | -17 | 29 | 6 | 3 |  | Pink | Auditory Network |
| 62 | 73 | -30 | -27 | 12 | 6 | 3 |  | Pink | Auditory Network |
| 63 | 74 | -41 | -75 | 26 | 6 | 4 |  | Red | Default Mode Network |
| 64 | 75 | 6 | 67 | -4 | 6 | 4 |  | Red | Default Mode Network |
| 65 | 76 | 8 | 48 | -15 | 6 | 4 |  | Red | Default Mode Network |
| 66 | 77 | -13 | -40 | 1 | 6 | 4 |  | Red | Default Mode Network |
| 67 | 78 | -18 | 63 | -9 | 6 | 4 |  | Red | Default Mode Network |
| 68 | 79 | -46 | -61 | 21 | 6 | 4 |  | Red | Default Mode Network |
| 69 | 80 | 43 | -72 | 28 | 6 | 4 |  | Red | Default Mode Network |
| 70 | 81 | -44 | 12 | -34 | 6 | 4 |  | Red | Default Mode Network |
| 71 | 82 | 46 | 16 | -30 | 6 | 4 |  | Red | Default Mode Network |
| 72 | 83 | -68 | -23 | -16 | 6 | 4 |  | Red | Default Mode Network |
| 73 | 86 | -44 | -65 | 35 | 6 | 4 |  | Red | Default Mode Network |
| 74 | 87 | -39 | -75 | 44 | 6 | 4 |  | Red | Default Mode Network |
| 75 | 88 | -7 | -55 | 27 | 6 | 4 |  | Red | Default Mode Network |
| 76 | 89 | 6 | -59 | 35 | 6 | 4 |  | Red | Default Mode Network |
| 77 | 90 | -11 | -56 | 16 | 6 | 4 |  | Red | Default Mode Network |
| 78 | 91 | -3 | -49 | 13 | 6 | 4 |  | Red | Default Mode Network |
| 79 | 92 | 8 | -48 | 31 | 6 | 4 |  | Red | Default Mode Network |
| 80 | 93 | 15 | -63 | 26 | 6 | 4 |  | Red | Default Mode Network |
| 81 | 94 | -2 | -37 | 44 | 6 | 4 |  | Red | Default Mode Network |
| 82 | 95 | 11 | -54 | 17 | 6 | 4 |  | Red | Default Mode Network |
| 83 | 96 | 52 | -59 | 36 | 6 | 4 |  | Red | Default Mode Network |
| 84 | 97 | 23 | 33 | 48 | 6 | 4 |  | Red | Default Mode Network |
| 85 | 98 | -10 | 39 | 52 | 6 | 4 |  | Red | Default Mode Network |
| 86 | 99 | -16 | 29 | 53 | 6 | 4 |  | Red | Default Mode Network |
| 87 | 100 | -35 | 20 | 51 | 6 | 4 |  | Red | Default Mode Network |
| 88 | 101 | 22 | 39 | 39 | 6 | 4 |  | Red | Default Mode Network |
| 89 | 102 | 13 | 55 | 38 | 6 | 4 |  | Red | Default Mode Network |
| 90 | 103 | -10 | 55 | 39 | 6 | 4 |  | Red | Default Mode Network |
| 91 | 104 | -20 | 45 | 39 | 6 | 4 |  | Red | Default Mode Network |
| 92 | 105 | 6 | 54 | 16 | 6 | 4 |  | Red | Default Mode Network |
| 93 | 106 | 6 | 64 | 22 | 6 | 4 |  | Red | Default Mode Network |
| 94 | 107 | -7 | 51 | -1 | 6 | 4 |  | Red | Default Mode Network |
| 95 | 108 | 9 | 54 | 3 | 6 | 4 |  | Red | Default Mode Network |
| 96 | 109 | -3 | 44 | -9 | 6 | 4 |  | Red | Default Mode Network |
| 97 | 110 | 8 | 42 | -5 | 6 | 4 |  | Red | Default Mode Network |
| 98 | 111 | -11 | 45 | 8 | 6 | 4 |  | Red | Default Mode Network |
| 99 | 112 | -2 | 38 | 36 | 6 | 4 |  | Red | Default Mode Network |
| 100 | 113 | -3 | 42 | 16 | 6 | 4 |  | Red | Default Mode Network |
| 101 | 114 | -20 | 64 | 19 | 6 | 4 |  | Red | Default Mode Network |
| 102 | 115 | -8 | 48 | 23 | 6 | 4 |  | Red | Default Mode Network |
| 103 | 116 | 65 | -12 | -19 | 6 | 4 |  | Red | Default Mode Network |
| 104 | 117 | -56 | -13 | -10 | 6 | 4 |  | Red | Default Mode Network |
| 105 | 118 | -58 | -30 | -4 | 6 | 4 |  | Red | Default Mode Network |
| 106 | 119 | 65 | -31 | -9 | 6 | 4 |  | Red | Default Mode Network |
| 107 | 120 | -68 | -41 | -5 | 6 | 4 |  | Red | Default Mode Network |
| 108 | 121 | 13 | 30 | 59 | 6 | 4 |  | Red | Default Mode Network |
| 109 | 122 | 12 | 36 | 20 | 6 | 4 |  | Red | Default Mode Network |
| 110 | 123 | 52 | -2 | -16 | 6 | 4 |  | Red | Default Mode Network |
| 111 | 124 | -26 | -40 | -8 | 6 | 4 |  | Red | Default Mode Network |
| 112 | 125 | 27 | -37 | -13 | 6 | 4 |  | Red | Default Mode Network |
| 113 | 126 | -34 | -38 | -16 | 6 | 4 |  | Red | Default Mode Network |
| 114 | 127 | 28 | -77 | -32 | 6 | 4 |  | Red | Default Mode Network |
| 115 | 128 | 52 | 7 | -30 | 6 | 4 |  | Red | Default Mode Network |
| 116 | 129 | -53 | 3 | -27 | 6 | 4 |  | Red | Default Mode Network |
| 117 | 130 | 47 | -50 | 29 | 6 | 4 |  | Red | Default Mode Network |
| 118 | 131 | -49 | -42 | 1 | 6 | 4 |  | Red | Default Mode Network |
| 119 | 137 | -46 | 31 | -13 | 6 | 4 |  | Red | Default Mode Network |
| 120 | 139 | 49 | 35 | -12 | 6 | 4 |  | Red | Default Mode Network |
| 121 | 143 | 18 | -47 | -10 | 6 | 5 |  | Blue | Visual Network |
| 122 | 144 | 40 | -72 | 14 | 6 | 5 |  | Blue | Visual Network |
| 123 | 145 | 8 | -72 | 11 | 6 | 5 |  | Blue | Visual Network |
| 124 | 146 | -8 | -81 | 7 | 6 | 5 |  | Blue | Visual Network |
| 125 | 147 | -28 | -79 | 19 | 6 | 5 |  | Blue | Visual Network |
| 126 | 148 | 20 | -66 | 2 | 6 | 5 |  | Blue | Visual Network |
| 127 | 149 | -24 | -91 | 19 | 6 | 5 |  | Blue | Visual Network |
| 128 | 150 | 27 | -59 | -9 | 6 | 5 |  | Blue | Visual Network |
| 129 | 151 | -15 | -72 | -8 | 6 | 5 |  | Blue | Visual Network |
| 130 | 152 | -18 | -68 | 5 | 6 | 5 |  | Blue | Visual Network |
| 131 | 153 | 43 | -78 | -12 | 6 | 5 |  | Blue | Visual Network |
| 132 | 154 | -47 | -76 | -10 | 6 | 5 |  | Blue | Visual Network |
| 133 | 155 | -14 | -91 | 31 | 6 | 5 |  | Blue | Visual Network |
| 134 | 156 | 15 | -87 | 37 | 6 | 5 |  | Blue | Visual Network |
| 135 | 157 | 29 | -77 | 25 | 6 | 5 |  | Blue | Visual Network |
| 136 | 158 | 20 | -86 | -2 | 6 | 5 |  | Blue | Visual Network |
| 137 | 159 | 15 | -77 | 31 | 6 | 5 |  | Blue | Visual Network |
| 138 | 160 | -16 | -52 | -1 | 6 | 5 |  | Blue | Visual Network |
| 139 | 161 | 42 | -66 | -8 | 6 | 5 |  | Blue | Visual Network |
| 140 | 162 | 24 | -87 | 24 | 6 | 5 |  | Blue | Visual Network |
| 141 | 163 | 6 | -72 | 24 | 6 | 5 |  | Blue | Visual Network |
| 142 | 164 | -42 | -74 | 0 | 6 | 5 |  | Blue | Visual Network |
| 143 | 165 | 26 | -79 | -16 | 6 | 5 |  | Blue | Visual Network |
| 144 | 166 | -16 | -77 | 34 | 6 | 5 |  | Blue | Visual Network |
| 145 | 167 | -3 | -81 | 21 | 6 | 5 |  | Blue | Visual Network |
| 146 | 168 | -40 | -88 | -6 | 6 | 5 |  | Blue | Visual Network |
| 147 | 169 | 37 | -84 | 13 | 6 | 5 |  | Blue | Visual Network |
| 148 | 170 | 6 | -81 | 6 | 6 | 5 |  | Blue | Visual Network |
| 149 | 171 | -26 | -90 | 3 | 6 | 5 |  | Blue | Visual Network |
| 150 | 172 | -33 | -79 | -13 | 6 | 5 |  | Blue | Visual Network |
| 151 | 173 | 37 | -81 | 1 | 6 | 5 |  | Blue | Visual Network |
| 152 | 174 | -44 | 2 | 46 | 6 | 6 |  | Yellow | Fronto-parietal Task Control Network |
| 153 | 175 | 48 | 25 | 27 | 6 | 6 |  | Yellow | Fronto-parietal Task Control Network |
| 154 | 176 | -47 | 11 | 23 | 6 | 6 |  | Yellow | Fronto-parietal Task Control Network |
| 155 | 177 | -53 | -49 | 43 | 6 | 6 |  | Yellow | Fronto-parietal Task Control Network |
| 156 | 178 | -23 | 11 | 64 | 6 | 6 |  | Yellow | Fronto-parietal Task Control Network |
| 157 | 179 | 58 | -53 | -14 | 6 | 6 |  | Yellow | Fronto-parietal Task Control Network |
| 158 | 180 | 24 | 45 | -15 | 6 | 6 |  | Yellow | Fronto-parietal Task Control Network |
| 159 | 181 | 34 | 54 | -13 | 6 | 6 |  | Yellow | Fronto-parietal Task Control Network |
| 160 | 186 | 47 | 10 | 33 | 6 | 6 |  | Yellow | Fronto-parietal Task Control Network |
| 161 | 187 | -41 | 6 | 33 | 6 | 6 |  | Yellow | Fronto-parietal Task Control Network |
| 162 | 188 | -42 | 38 | 21 | 6 | 6 |  | Yellow | Fronto-parietal Task Control Network |
| 163 | 189 | 38 | 43 | 15 | 6 | 6 |  | Yellow | Fronto-parietal Task Control Network |
| 164 | 190 | 49 | -42 | 45 | 6 | 6 |  | Yellow | Fronto-parietal Task Control Network |
| 165 | 191 | -28 | -58 | 48 | 6 | 6 |  | Yellow | Fronto-parietal Task Control Network |
| 166 | 192 | 44 | -53 | 47 | 6 | 6 |  | Yellow | Fronto-parietal Task Control Network |
| 167 | 193 | 32 | 14 | 56 | 6 | 6 |  | Yellow | Fronto-parietal Task Control Network |
| 168 | 194 | 37 | -65 | 40 | 6 | 6 |  | Yellow | Fronto-parietal Task Control Network |
| 169 | 195 | -42 | -55 | 45 | 6 | 6 |  | Yellow | Fronto-parietal Task Control Network |
| 170 | 196 | 40 | 18 | 40 | 6 | 6 |  | Yellow | Fronto-parietal Task Control Network |
| 171 | 197 | -34 | 55 | 4 | 6 | 6 |  | Yellow | Fronto-parietal Task Control Network |
| 172 | 198 | -42 | 45 | -2 | 6 | 6 |  | Yellow | Fronto-parietal Task Control Network |
| 173 | 199 | 33 | -53 | 44 | 6 | 6 |  | Yellow | Fronto-parietal Task Control Network |
| 174 | 200 | 43 | 49 | -2 | 6 | 6 |  | Yellow | Fronto-parietal Task Control Network |
| 175 | 201 | -42 | 25 | 30 | 6 | 6 |  | Yellow | Fronto-parietal Task Control Network |
| 176 | 202 | -3 | 26 | 44 | 6 | 6 |  | Yellow | Fronto-parietal Task Control Network |
| 177 | 203 | 11 | -39 | 50 | 6 | 7 |  | Orange | Salience Network |
| 178 | 204 | 55 | -45 | 37 | 6 | 7 |  | Orange | Salience Network |
| 179 | 205 | 42 | 0 | 47 | 6 | 7 |  | Orange | Salience Network |
| 180 | 206 | 31 | 33 | 26 | 6 | 7 |  | Orange | Salience Network |
| 181 | 207 | 48 | 22 | 10 | 6 | 7 |  | Orange | Salience Network |
| 182 | 208 | -35 | 20 | 0 | 6 | 7 |  | Orange | Salience Network |
| 183 | 209 | 36 | 22 | 3 | 6 | 7 |  | Orange | Salience Network |
| 184 | 210 | 37 | 32 | -2 | 6 | 7 |  | Orange | Salience Network |
| 185 | 211 | 34 | 16 | -8 | 6 | 7 |  | Orange | Salience Network |
| 186 | 212 | -11 | 26 | 25 | 6 | 7 |  | Orange | Salience Network |
| 187 | 213 | -1 | 15 | 44 | 6 | 7 |  | Orange | Salience Network |
| 188 | 214 | -28 | 52 | 21 | 6 | 7 |  | Orange | Salience Network |
| 189 | 215 | 0 | 30 | 27 | 6 | 7 |  | Orange | Salience Network |
| 190 | 216 | 5 | 23 | 37 | 6 | 7 |  | Orange | Salience Network |
| 191 | 217 | 10 | 22 | 27 | 6 | 7 |  | Orange | Salience Network |
| 192 | 218 | 31 | 56 | 14 | 6 | 7 |  | Orange | Salience Network |
| 193 | 219 | 26 | 50 | 27 | 6 | 7 |  | Orange | Salience Network |
| 194 | 220 | -39 | 51 | 17 | 6 | 7 |  | Orange | Salience Network |
| 195 | 222 | 6 | -24 | 0 | 6 | 8 |  | Brown | Subcortical Network |
| 196 | 223 | -2 | -13 | 12 | 6 | 8 |  | Brown | Subcortical Network |
| 197 | 224 | -10 | -18 | 7 | 6 | 8 |  | Brown | Subcortical Network |
| 198 | 225 | 12 | -17 | 8 | 6 | 8 |  | Brown | Subcortical Network |
| 199 | 226 | -5 | -28 | -4 | 6 | 8 |  | Brown | Subcortical Network |
| 200 | 227 | -22 | 7 | -5 | 6 | 8 |  | Brown | Subcortical Network |
| 201 | 228 | -15 | 4 | 8 | 6 | 8 |  | Brown | Subcortical Network |
| 202 | 229 | 31 | -14 | 2 | 6 | 8 |  | Brown | Subcortical Network |
| 203 | 230 | 23 | 10 | 1 | 6 | 8 |  | Brown | Subcortical Network |
| 204 | 231 | 29 | 1 | 4 | 6 | 8 |  | Brown | Subcortical Network |
| 205 | 232 | -31 | -11 | 0 | 6 | 8 |  | Brown | Subcortical Network |
| 206 | 233 | 15 | 5 | 7 | 6 | 8 |  | Brown | Subcortical Network |
| 207 | 234 | 9 | -4 | 6 | 6 | 8 |  | Brown | Subcortical Network |
| 208 | 138 | -10 | 11 | 67 | 6 | 9 |  | Green | Ventral/Dorsal Attention Network |
| 209 | 235 | 54 | -43 | 22 | 6 | 9 |  | Green | Ventral/Dorsal Attention Network |
| 210 | 236 | -56 | -50 | 10 | 6 | 9 |  | Green | Ventral/Dorsal Attention Network |
| 211 | 237 | -55 | -40 | 14 | 6 | 9 |  | Green | Ventral/Dorsal Attention Network |
| 212 | 238 | 52 | -33 | 8 | 6 | 9 |  | Green | Ventral/Dorsal Attention Network |
| 213 | 239 | 51 | -29 | -4 | 6 | 9 |  | Green | Ventral/Dorsal Attention Network |
| 214 | 240 | 56 | -46 | 11 | 6 | 9 |  | Green | Ventral/Dorsal Attention Network |
| 215 | 241 | 53 | 33 | 1 | 6 | 9 |  | Green | Ventral/Dorsal Attention Network |
| 216 | 242 | -49 | 25 | -1 | 6 | 9 |  | Green | Ventral/Dorsal Attention Network |
| 217 | 251 | 10 | -62 | 61 | 6 | 9 |  | Green | Ventral/Dorsal Attention Network |
| 218 | 252 | -52 | -63 | 5 | 6 | 9 |  | Green | Ventral/Dorsal Attention Network |
| 219 | 256 | 22 | -65 | 48 | 6 | 9 |  | Green | Ventral/Dorsal Attention Network |
| 220 | 257 | 46 | -59 | 4 | 6 | 9 |  | Green | Ventral/Dorsal Attention Network |
| 221 | 258 | 25 | -58 | 60 | 6 | 9 |  | Green | Ventral/Dorsal Attention Network |
| 222 | 259 | -33 | -46 | 47 | 6 | 9 |  | Green | Ventral/Dorsal Attention Network |
| 223 | 260 | -27 | -71 | 37 | 6 | 9 |  | Green | Ventral/Dorsal Attention Network |
| 224 | 261 | -32 | -1 | 54 | 6 | 9 |  | Green | Ventral/Dorsal Attention Network |
| 225 | 262 | -42 | -60 | -9 | 6 | 9 |  | Green | Ventral/Dorsal Attention Network |
| 226 | 263 | -17 | -59 | 64 | 6 | 9 |  | Green | Ventral/Dorsal Attention Network |
| 227 | 264 | 29 | -5 | 54 | 6 | 9 |  | Green | Ventral/Dorsal Attention Network |

**Supplementary Table S2. Detailed P values of between-group differences of inter-RSN dynamics.**

| **FOI-1** | **SMN** | **CON** | **AN** | **DMN** | **VN** | **FPN** | **SN** | **SCN** | **VDN** |
| --- | --- | --- | --- | --- | --- | --- | --- | --- | --- |
| **SMN** |  | P>0.05 | P>0.05 | P=0.008* | P=0.001* | P>0.05 | P>0.05 | P>0.05 | P>0.05 |
| **CON** |  |  | P>0.05 | P=0.005* | P=0.003* | P>0.05 | P>0.05 | P>0.05 | P=0.002* |
| **AN** |  |  |  | P>0.05 | P>0.05 | P>0.05 | P>0.05 | P>0.05 | P=0.001* |
| **DMN** |  |  |  |  | P>0.05 | P>0.05 | P=0.002* | P>0.05 | P=0.002* |
| **VN** |  |  |  |  |  | P=0.002* | P=0.002* | P>0.05 | P=0.010* |
| **FPN** |  |  |  |  |  |  | P>0.05 | P>0.05 | P=0.002* |
| **SN** |  |  |  |  |  |  |  | P>0.05 | P>0.05 |
| **SCN** |  |  |  |  |  |  |  |  | P>0.05 |
| **VDN** |  |  |  |  |  |  |  |  |  |

| **FOI-2** | **SMN** | **CON** | **AN** | **DMN** | **VN** | **FPN** | **SN** | **SCN** | **VDN** |
| --- | --- | --- | --- | --- | --- | --- | --- | --- | --- |
| **SMN** |  | P>0.05 | P>0.05 | P>0.05 | P>0.05 | P>0.05 | P>0.05 | P>0.05 | P>0.05 |
| **CON** |  |  | P>0.05 | P>0.05 | P=0.005* | P>0.05 | P>0.05 | P>0.05 | P=0.007* |
| **AN** |  |  |  | P=0.009* | P=0.006* | P>0.05 | P>0.05 | P>0.05 | P=0.001* |
| **DMN** |  |  |  |  | P>0.05 | P=0.001* | P=0.002* | P>0.05 | P=0.001* |
| **VN** |  |  |  |  |  | P>0.05 | P=0.006* | P>0.05 | P=0.010* |
| **FPN** |  |  |  |  |  |  | P>0.05 | P>0.05 | P=0.002* |
| **SN** |  |  |  |  |  |  |  | P>0.05 | P=0.010* |
| **SCN** |  |  |  |  |  |  |  |  | P=0.003* |
| **VDN** |  |  |  |  |  |  |  |  |  |

| **FOI-3** | **SMN** | **CON** | **AN** | **DMN** | **VN** | **FPN** | **SN** | **SCN** | **VDN** |
| --- | --- | --- | --- | --- | --- | --- | --- | --- | --- |
| **SMN** |  | P>0.05 | P>0.05 | P>0.05 | P>0.05 | P>0.05 | P=0.004* | P>0.05 | P>0.05 |
| **CON** |  |  | P>0.05 | P>0.05 | P>0.05 | P>0.05 | P>0.05 | P>0.05 | P>0.05 |
| **AN** |  |  |  | P>0.05 | P>0.05 | P>0.05 | P>0.05 | P>0.05 | P>0.05 |
| **DMN** |  |  |  |  | P>0.05 | P>0.05 | P>0.05 | P>0.05 | P=0.001* |
| **VN** |  |  |  |  |  | P>0.05 | P=0.004* | P>0.05 | P>0.05 |
| **FPN** |  |  |  |  |  |  | P>0.05 | P>0.05 | P>0.05 |
| **SN** |  |  |  |  |  |  |  | P>0.05 | P=0.001* |
| **SCN** |  |  |  |  |  |  |  |  | P=0.003* |
| **VDN** |  |  |  |  |  |  |  |  |  |

| **FOI-4** | **SMN** | **CON** | **AN** | **DMN** | **VN** | **FPN** | **SN** | **SCN** | **VDN** |
| --- | --- | --- | --- | --- | --- | --- | --- | --- | --- |
| **SMN** |  | P>0.05 | P>0.05 | P=0.002* | P>0.05 | P>0.05 | P>0.05 | P=0.006* | P>0.05 |
| **CON** |  |  | P>0.05 | P=0.007* | P>0.05 | P>0.05 | P>0.05 | P>0.05 | P>0.05 |
| **AN** |  |  |  | P>0.05 | P>0.05 | P>0.05 | P>0.05 | P>0.05 | P>0.05 |
| **DMN** |  |  |  |  | P=0.003* | P>0.05 | P=0.007* | P=0.003* | P>0.05 |
| **VN** |  |  |  |  |  | P=0.004* | P=0.005* | P=0.006* | P>0.05 |
| **FPN** |  |  |  |  |  |  | P>0.05 | P=0.002* | P>0.05 |
| **SN** |  |  |  |  |  |  |  | P=0.002* | P>0.05 |
| **SCN** |  |  |  |  |  |  |  |  | P>0.05 |
| **VDN** |  |  |  |  |  |  |  |  |  |

Asterisks indicate a significant group difference with two sample *t* test, significant level was set at *P* < 0.05 for Bonferroni corrected.
